# Sustainable Green Lightweight Concrete Containing Plastic-Based Green Lightweight Aggregate

**DOI:** 10.3390/ma14123304

**Published:** 2021-06-15

**Authors:** Fahad K. Alqahtani

**Affiliations:** Department of Civil Engineering, College of Engineering, King Saud University, P.O. Box 800, Riyadh 11421, Saudi Arabia; bfahad@ksu.edu.sa

**Keywords:** plastic waste, additives, plastic-based green lightweight aggregate, green lightweight concrete, chloride penetration

## Abstract

Nowadays the environment and its natural resources face many issues, related to the depletion of natural resources beside the increase in environmental pollution resulted from uncontrolled plastic waste disposal. Therefore, it is important to find effective and feasible solutions to utilize these wastes, such as using them to produce environmentally friendly green concrete. In this study, plastic-based green lightweight aggregates (PGLAs) containing PET plastic waste and by product additives were developed, and their subsequent physical and mechanical properties were compared with those of reference aggregates. Then, green lightweight aggregate concrete mixes (GLACs) were produced at 100% replacement of normal weight and lightweight coarse aggregate with developed PGLAs; and their fresh, hardened, microscopic and durability-related properties were compared to those of control mixes. Study results revealed that the unit weight of PGLAs were 21% to 29% less than that of normal coarse aggregate. Additionally, PGLAs had low water absorption that varied between 1.2% and 1.6%. The developed aggregates had 45% higher strength compared to that of lightweight coarse aggregate. Study results confirmed that structural green lightweight aggregate concretes (GLACs), that satisfied the dry density, compressive and splitting tensile strength requirements specified in ASTM C330, were feasibly produced. Finally, GLACs had low-to-moderate chloride penetration in accordance with ASTM C1202, thus it can be used in those areas exposed to the risk of chloride attack.

## 1. Introduction

Demand of concrete has boomed up to 30 billion ton/year in the construction industry [1]. Currently there is great interest in reducing the life cycle cost (LCC) of concrete. This can be achieved through the use of lightweight concrete, thus obtaining fewer material quantities for the building elements due to the lower dead weight of the concrete. The lightweight concrete has a number of advantages, including less unit weight, compressive strength ranging from 7 to 25 MPa, good insulation properties along with low handling and transportation cost, which ultimately reduces the construction cost. One of the approaches to produce lightweight concrete is by using lightweight aggregates, such as lightweight aggregates formed from plastic waste products.

On the other hand, the continuous production and use of plastic products have resulted in huge quantities of plastic (i.e., 6300 Mt in 2017) [2], accumulated in stockpiles (only 9% recycled) causing negative health, environment and economic impacts. Beside plastic recycling, attempts were made to encourage the use of plastic waste in various application, such as in aggregate and concrete production. Incorporating plastic waste (i.e., polyethylene terephthalate—PET) either directly or indirectly as (coarse and fine) aggregate in concrete production not only reduces the burden on landfill dumping sites, but also aids economical concrete construction. The life cycle cost of plastic-based aggregate concrete is significantly reduced compared to that of normal concrete.

Previously, natural coarse aggregate (CA) and fine aggregate (FA) were replaced by light weight plastic aggregates for production of lightweight concrete (LWC). For instance, LWC produced by replacing 5% to 75% FA with shredded PET bottles [3,4,5,6,7]. Similarly, CA and FA were replaced from 5% to 15% by shredded PET plastic for LWC production [8,9,10,11].

Besides decreasing the weight of the concrete, plastic aggregates significantly reduce the slump of concrete. For instance, a 95% concrete slump reduction was noticed by replacing 20% FA with plastic waste [12]; while another study [13] reported a decrease in the slump varied that from 42% to 73% at 20% replacement of FA with PET. This linked to the friction of the non-uniform and high surface area of shaped shredded plastic [6,12,13,14]. On the other hand, synthetic aggregates, for example, waste plastic lightweight aggregate (WPLA), having spherical shape, smooth texture and less water absorption properties increased the workability of concrete in the case of replacing FA with WPLA [15,16].

Moreover, fresh density of LWC containing plastic particles is governed by plastic proportions and type. For instance, 80% and 55% replacement of CA and total aggregates with expanded polystyrene (EPS) reduced the fresh density by 40% and 64%, respectively, [17,18]. Similarly, replacing 20% of FA with plastic waste reduced fresh density by 9% [12]. The reduction in fresh density is attributed to the light weight of plastic as compared to natural aggregates [6,8,10,11,13,14,19]. Likewise, the dry density of LWC produced by replacing 40% to 80% CA with plastic waste reduced dry density from 11% to 68% [18,20,21,22,23,24,25]. As similarly reported in fresh density, the reduction in dry density is governed by the light weight of plastic, so the dry density decreases when plastic waste increases [15,16,26,27,28].

The compressive strength of plastic-based lightweight concrete decrease significantly due to replacement of high compressive strength conventional CA and FA with plastic aggregates. For instance, 62% to 82% reduction in compressive strength was noticed by replacing 15% to 80% of CA with plastic (i.e., EPS, high density polyethylene-HDPE, PET, polyurethane—PUR foam and ethylene vinyl acetate—EVA) [10,18,19,20,22,23,24,25]. Similarly, 75% replacement of FA with plastic (i.e., WPLA) reduced compressive strength by 33%, 33% and 31% at water cement ratio W/C of 0.45, 0.49 and 0.53, respectively [15,16]. In case of using synthetic plastic-based aggregate, 100% replacement of CA with synthetic plastic (i.e., SLA) caused 43% to 72% reduction of compressive strength, as compared to control samples [26,28,29]. The prime factor of reducing compressive strength is the weak interfacial transition zone (ITZ) between cement and the plastic particles [5,6,13,14,20,24,30]. Moreover, the hydrophobic nature of plastic hinders the hydration process of LWC, resulting in compressive strength reduction [13,30].

In addition, previous studies reported a reduction in splitting tensile strength ranging from 26% to 38%, and from 37% to 40%, by replacing CA at 100% and FA at 75% with plastic-based aggregate (i.e., SLA or WPLA), respectively. Similarly, a reduction in flexural strength that varied between 50% to 59% was reported previously at different replacement levels, which ranged from 15% to 50% of CA with plastic particles [10,23]. The reduction in splitting and flexural strength can be explained by the differences in physical and mechanical properties, such as shape, texture and strength, of plastic aggregate and that of conventional aggregate [6,10,30,31]. In addition, to the weak bonding between plastic particles and cement paste, similarly as reported in compressive strength [9,30].

In the study conducted by Marzouk et al. [32], scanning electron microscope (SEM) images of LWC produced by 30%, 50% and 100% replacement of FA with PET were presented. The study results showed that the composites were highly compacted up to 50% replacement, while it was porous at 100% replacement levels. The internal structure variations (i.e., high porosity) is linked with lower strength characteristics and bulk density of the mix [32].

On the other hand, the durability and durability-related properties for the concrete made with plastic has received a little attention. According to Kou et al. (2009) [30], the chloride permeability decreased by 36% and 43% at 45% and 37% replacement of FA and TA with polyvinyl chloride (PVC) and EPS, respectively.

Plastic-based lightweight aggregate concrete is predominately produced by direct incorporation; however, indirect replacement, where synthetic aggregate was produced prior to its implantation in concrete, is less focused. The synthetic aggregates, such as polyethylene terephthalate (PET) and fillers (i.e., fly ash), are less covered [15,16,26,28,29,33,34,35,36]. For instance, PET with granulated blast furnace slag (GBFS) and river sand powder were used to develop the WPLA [15,16]. The authors reported that the WPLA particles possessed a rounded shape and smooth surface texture. Moreover, the produced WPLA had density and water absorption of 1390 kg/m^3^ and 0%, respectively [15,16].

Furthermore, lightweight aggregates were produced by melting mixed plastic comprised of 85% PET and 15% polypropylene (PP) [37]. The produced aggregate had a smooth surface texture with angular particle shape. Additionally, the density and water absorption of the produced aggregate was 1.2 g/cm^3^ and 0% [37]. In another study [38], the synthetic aggregate (i.e., fly ash aggregate—FAA) was successfully produced by sintering fly ash. The particles of produced aggregate possess angularity in their shape and roughness in their surface texture. The water absorption of FAA was 60% lower than that of control mix [38].

The effect of incorporating shredded or virgin plastic, as it is in concrete, has extensively been studied over the last few decades. However, less research work has been carried out to use plastic-produced synthetic aggregate in concrete. Thus, the current study focuses on the formulation of a plastic-based green lightweight aggregate (PGLA) manufactured using PET plastic waste and mineral additives. In addition, a green lightweight aggregate concrete (GLAC), comprising 100% manufactured synthetic plastic aggregates as a replacement of normal weight and lightweight aggregate, was also produced. Comparison of physical and mechanical properties for the produced green aggregates with the reference one was also made. Likewise, all the major properties including durability properties of GLACs were also examined and equated to the concrete made with conventional normal weight and lightweight aggregates.

## 2. Materials and Methods

### 2.1. Material Used

In this investigation, locally available ordinary Portland cement that complies with the standard specifications (i.e., ASTM C150) was used. The specific gravity, initial setting time, final setting time and consistency were 3.15, 45 min, 135 min and 23.5%, respectively. The coarse aggregate was used at various levels, whilst the type and proportions of ordinary Portland cement, fine aggregates and water were constant throughout the investigation.

**Figure 1:**
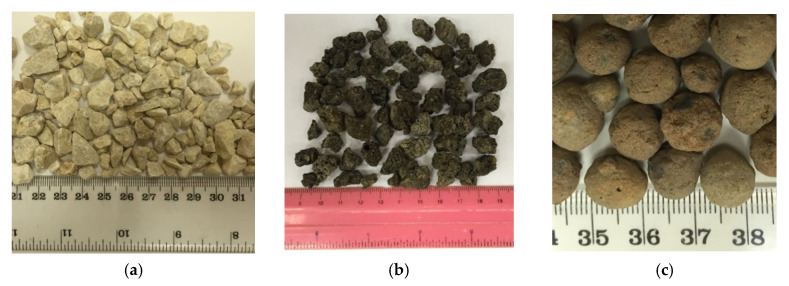
Reference coarse aggregates used: normal weight coarse aggregate (NCA) (**a**); volcanic lightweight coarse aggregate (VLA) (**b**) and lytag coarse aggregate (LYA) (**c**).

Four different types of coarse aggregates, comprising normal weight aggregate (NCA), volcanic lightweight aggregate (VLA), Lytage aggregate (LYA) and plastic-based green lightweight aggregate (PGLA), were used in this study. The size, shape and texture of NCA, VLA and LYA are shown below in Figure 1. However, the development process of PGLAs together with the properties are described in detail in Section 3 and Section 4, respectively.

The normal weight coarse aggregate (NCA) collected from local sources in Riyadh, Saudi Arabia was used in experimental work to produce a normal weight concrete (NCAC) control mix. Additionally, a local volcanic lightweight aggregate (VLA) was used for the reference mix of local lightweight concrete (VLAC). The VLA used in this study was collected from the western side of Saudi Arabia. The LYA was supplied by Lytag International Ltd. in York, UK. The main material in this investigation was plastic-based green lightweight aggregate (PGLA), which was developed using PET plastic waste and additives, discussed in detail in Section 3. The green lightweight aggregate concrete (GLAC) was produced using PGLA at full replacement of NCA and VLA.

**Figure 2:**
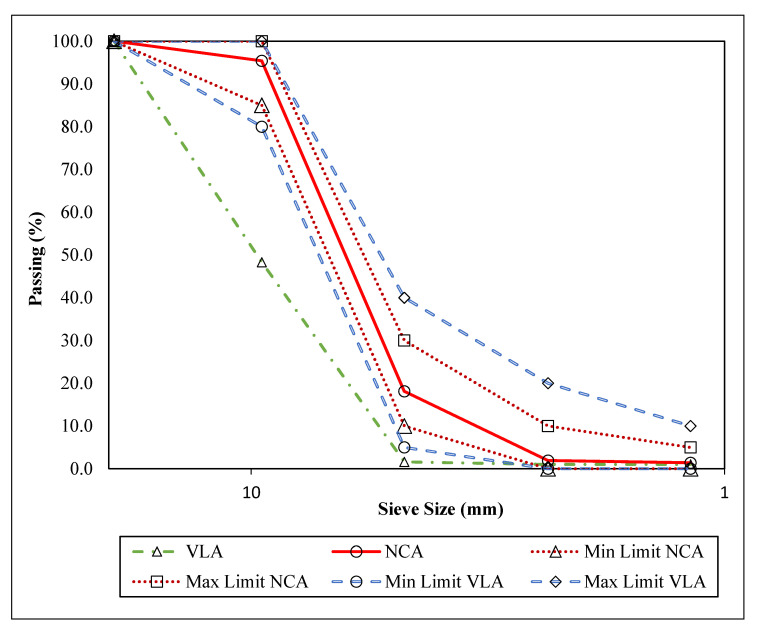
Grading curve of normal weight coarse (NCA) and volcanic lightweight coarse (VLA) aggregate.

The physical properties of NCA and VLA used in this study are given in Table 1; while PGLA properties are discussed in Section 4. The specific gravity and waster absorption, unit weight and voids for the reference aggregate were conducted according to test producers specified in ASTM C127-15 and ASTM C29/C29M-16. Moreover, the particle size distribution curves for NCA and VLA are presented in Figure 2, along with their standard limits for normal weight and lightweight aggregates. However, the particle size distribution of LYA was made in the lab according to the grading of conventional lightweight aggregate, since it was supplied in a single grade by its manufacturer.

**Figure 3:**
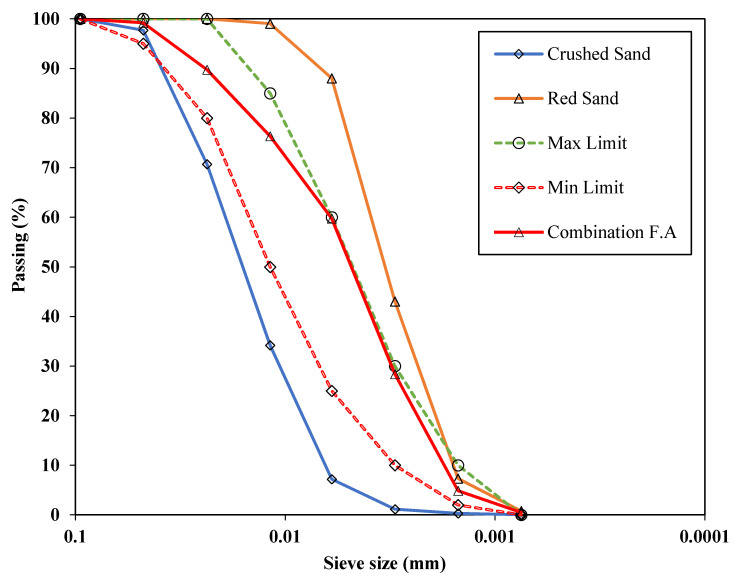
Particle size distribution of fine aggregate used in this study.

However, the normal weight fine aggregate was used in the current study which comprised of 35% crushed sand and 65% red sand. The grading curves are shown [39,40] in Figure 3. Table 1 shows the major properties for these sands.

### 2.2. Mix Design Procedure and Test Conducted

The volume method was used in the current study for selecting the mix proportions in accordance with standard mix procedures specified in [41]. A constant water-to-cement ratio of 0.5 was used for all the concrete series. Three different concrete types with a total of 5 concrete series were prepared. For example, NCAC and VLAC refer to concrete with 100% of normal weight and lightweight aggregates, while GLAC2 stands for green concrete containing plastic-based green lightweight aggregate (i.e., PGLA2). The detailed mix proportions for the said concrete series are shown in Table 2 for 1 m^3^. The sample designations are provided under the table.

In general, the lightweight aggregate standard described in the [42] was used to carry out the tests on aggregates. Table 3 shows the tests conducted for the different concrete series. All the mixing, casting and curing of concrete were carried out as per the procedures specified in [43]. An average of three samples for all the tested properties was used, except for the fresh properties, for which the average of two samples was used as per [43,44]. The average of three results yielded a variation within ±5% of the mean values.

## 3. Plastic-Based Green Lightweight Aggregate Production

**Figure 4:**
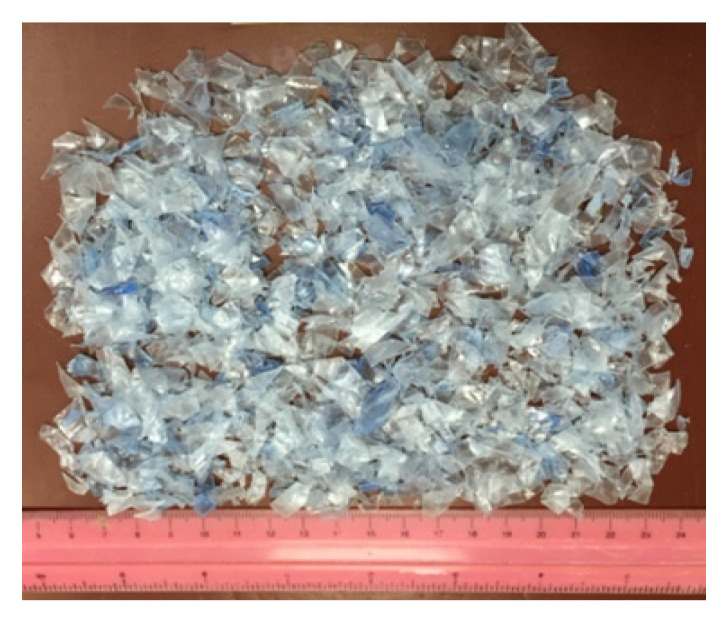
Polyethylene terephthalate (PET) plastics waste used in developing plastic based green lightweight aggregate (PGLA).

Due to the fact that not all plastic can be recycled, along with the differences in the physical, thermal and microstructure properties of plastic such as melting temperature [50,51], a polyethylene terephthalate (PET) was chosen amongst various recycled plastics, based on its bulk availability in the plastic production and waste stream, as extensively reported in the literature. The plastic polyethylene terephthalate (PET) used was supplied by a local recycling supplier and shredded to the desired size (see Figure 4) for the production of PGLA. The unit weight, water absorption and melting temperature of the PET used were 838 kg/m^3^, 0.10–0.20% and 285 °C, respectively.

**Figure 5:**
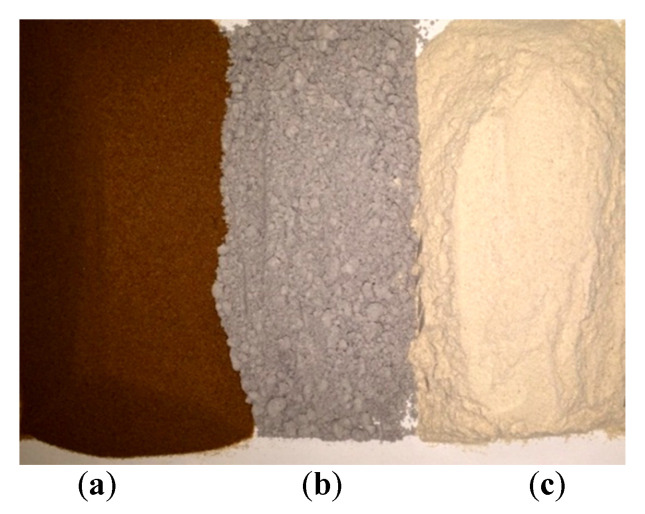
Different types of additives: dune dust additive (DDA) (**a**), fly ash additive (FAA) (**b**) and quarry dust additive (QDA) (**c**).

Similarly, three types of additives (see Figure 5), namely dune dust additive (DDA), fly ash additive (FAA) and quarry dust additive (QDA), were selected according to their local availability and based on their similarity with the major components in concrete.

The specific gravity, absorption and unit weight of DDA and QDA were examined according to ASTM C128 and ASTM C29. Whereas ASTM C618 was followed to analyze specific gravity of FAA. The specific gravity of FAA, QDA and DDA was 2.3, 2.71 and 2.62, respectively. While the water absorption of QDA and DDA was 1.52% and 0.28%, respectively. The particle size distribution of additives was performed using the laser particle size analyzer. The median size of FAA, QDA and DDA was 6.14, 19.27 and 186.37 µm, respectively, which revealed that FAA was the finest, while DDA was the coarsest among these additives.

The compression press was used in the manufacturing process of the plastic-based green aggregates. A homogenized mixture was formed, compressed, heated, melted and then cooled before grinding to give the final shape of the produced aggregates. The steps used in the production of these aggregates have already been patented under [52]. Applying the before mentioned method, three different aggregate types were produced using three different categories of additives (i.e., dune dust additive (DDA), flay ash additive (FAA) or quarry dust additive (QDA) with a ratio of 30:70 (plastic: additives) as shown in Table 4.

**Figure 6:**
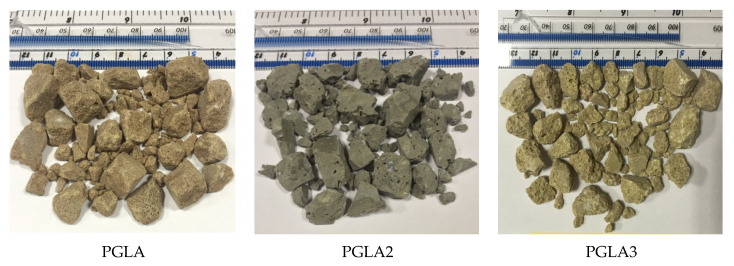
Different types of PGLAs developed using plastic waste and mineral additives.

Figure 6 shows the produced PGLA and it was observed that DDA, FAA and QDA produced brown, grey and yellow PGLA, respectively. The difference in the colour was principally due to the different colour of the additives added during the manufacturing of the different aggregate series.

In the subsequent sections, the physical, mechanical and morphology properties of the manufactured PGLA aggregates will be presented and also a comparison will be made with the NCA, VLA and LYA, along with similar studies conducted to produce plastic-based aggregates.

## 4. Plastic-Based Green Lightweight Aggregate Investigation

The developed PGLAs were investigated in terms of their physical, mechanical and morphological properties. The summary of the findings of these tests are presented in Table 5.

The optical microscope was used to evaluate the shape and surface textures of PGLA particles. The shape of the PGLAs developed with DDA, FAA and QDA was more angular with sharp edges. This can be explained by the high degree of stiffness of PGLA, since it breaks in the crushing phase of aggregate production, rather than shredding, as reported in similar plastic-based aggregate produced in the literature.

In addition, the surface textures of the PGLAs (Figure 6) varied from partially smooth to rough depending on the type of additives used. In general, the texture of PGLAs was smoother than those of plastic-based aggregate reported in the previous studies. This observation can be linked to the behavior of the PGLAs during the crushing, since other plastic-based aggregates were shredded instead of crushed, as mentioned in previous studies.

Therefore, it can be concluded that the PGLAs are comparable to that of NCA in terms of particle shape and texture. However, the PGLAs were entirely different to VLA and LYA. It was noticed that the additives type mainly governed the shape and texture of the plastic-based green lightweight aggregate. Furthermore, it was also observed that shape and texture of the SPGA series were not identical to each other, as well as that reported in the previous studies [15,16,26]. The differences in the experimental procedures and raw materials used for the manufacturing of PGLAs series, as compared to the previous studies, are mainly responsible for the variation in the shape and texture of produced aggregates.

**Figure 7:**
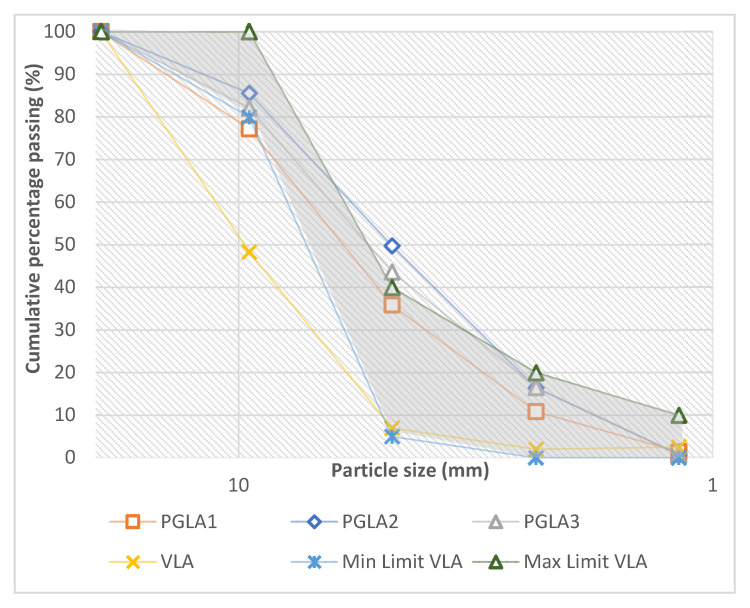
Particle size distribution curves for PGLAs and VLA.

**Figure 8:**
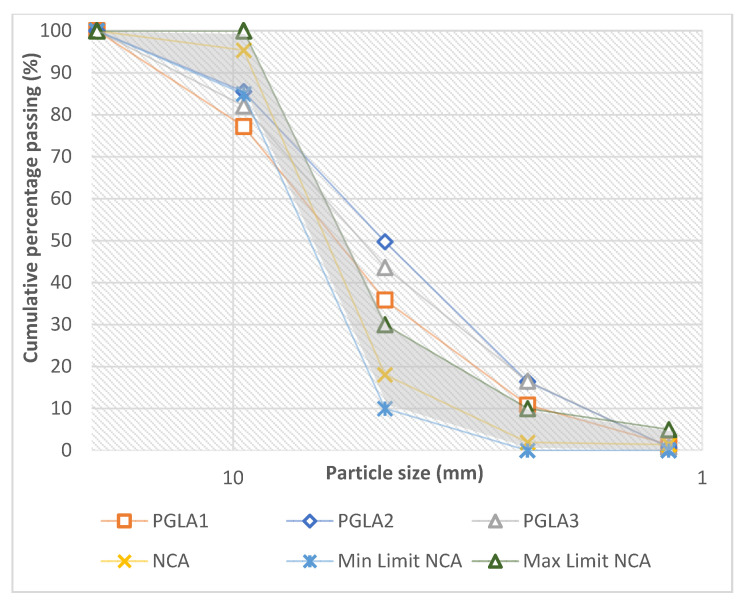
Particle size distribution curves for PGLAs and NCA.

The voids between particles of aggregates are controlled by the size and shape of the particles. For example, poorly graded aggregate exhibits a higher void percentage [53]. Consequently, more cement paste is needed, as the amount of cement paste influences by the percentage of voids [54]. Therefore, sieve analysis was conducted for PGLAs to develop the particle size distribution curves plotted in Figure 7, together with that for VLA and the lightweight standard limits. The particle size distribution curves of the PGLAs were also compared with those of NCA and the normal weight standard limits, as shown in Figure 8. As it can be seen in these figures, the nominal maximum size of the PGLAs, VLA and NCA is 10 mm.

As it can be seen in Figure 7, the tested aggregates were classified into the following categories as per the findings of the sieve analysis:

- Category 1—contains aggregates that diverged from the maximum limits of [42] for the lower sieve size (#4), as seen in PGLA2 and PGLA3. These aggregates deviated by 19% and 8%, respectively.
- Category 2—which diverged from the minimum limits of [42] for the upper sieve (#3/8). For instance, PGLA1 slightly deviated by 3.5%, while VLA significantly deviated by 39%.

However, comparing PGLAs with NCA (see Figure 8) revealed that the grading of PGLAs is poor compared with that of NCA and it does not fulfil the minimum and maximum grading requirements for the normal weight coarse aggregate. This trend is expected as the developed aggregate is lightweight not a normal weight.

These results showed that the PGLAs made with DDA were the coarsest, followed by those PGLAs made with QDA and, finally, the finest were the PGLAs made with FAA. This is accredited to the sizes of the additive particles themselves, since the median size of DDA was the largest, whereas FAA had the smallest particles size among all the additives. The void percentage of the PGLAs (see Table 5) was 13% to 34%, 34% to 50% and 15% to 36% lower than that of NCA, VLA and LYA, respectively. Additionally, the percentage of voids in the PGLAs is lower as compared to all reference aggregates, which proves that the aggregates’ particles in PGLAs have a better angularity, as reported earlier.

Moreover, Table 5 showed no major impact of PGLAs on the fineness modulus values. Findings show a reduction in fineness modulus ranging between 1.5% and 13%, as compared to NCA and VLA; with exception of PGLA1, which showed a marginal increase by 0.7%. Moreover, the reference lightweight aggregates (i.e., VLA) showed the highest fineness modulus as compared to all the aggregate types. These results indicate that PGLA1 (i.e., containing DDA) was the coarsest, while PGLA2 (i.e., containing DDA) was the finest. This is associated with the large size of the DDA particles in contrast with the sizes of FAA.

The specific gravity or density is a crucial property, as it is used to determine the aggregates’ volume in the mix. Additionally, controlling the density of concrete as aggregate represents almost 70% of the total volume of concrete’s components. Table 5 presents the bulk specific gravity of PGLAs in saturated surface dry (SSD) and oven dry (OD) states. As it can be seen in Table 5, the maximum dry density/specific gravity was achieved by PGLA1 (i.e., 70% DDA), because the DDA had the largest density among all additives. Additionally, the specific gravity of PGLAs increased from 26% to 38% and from 24% to 35% with respect to those of VLA and LYA, respectively. However, it was 24% to 31% less than that of NCA. Similarly, the dry density of PGLAs was 61% to 81% and 26% to 42% higher than those of VLA and LYA, respectively; while it was 21% to 29% less than that of NCA. Although the developed PGLAs showed higher specific gravity/density compared to reference lightweight aggregates, these results are comparable to those plastic-based aggregates developed in previous studies [26,27,55,56,57]. In those studies, the specific gravity ranged between 0.9 and 1.9 for different plastic-based aggregates.

The observed increase in specific gravity and density of PGLAs, as compared to that of VLA and LYA, is attributed to the least number of voids between PGLAs as compared to that in VLA and LYA (See Table 5). However, the reduction in PGLAs in comparison with NCA is linked with the lighter weight of the plastic and additives together with the increase in void percentages of PGLAs, as confirmed by the SEM images.

Furthermore, the concretes’ quality depends on the precise calculation of aggregates’ water absorption, because some concrete properties, such as porosity, cement hydration and slump, were significantly affected with the extra or shortage of water in the mix [57].

The results in Table 5 revealed a significant reduction in PGLAs’ water absorption, ranging from 90% to 92%, from 90% to 93% and from 6% to 18%, as compared to LYA, VLA and NCA, respectively; except PGLA3, which had a 13% increase compared to NCA. This substantial reduction in the water absorption of the PGLAs is due to the water repellent feature of plastic in the PGLAs matrix. These results suggest that the water absorption of the developed aggregate is less compared with those reported in literature [26,28,55,56]. In those studies, the aggregates’ water absorption ranged from 4.2% to 19.3%.

**Figure 9:**
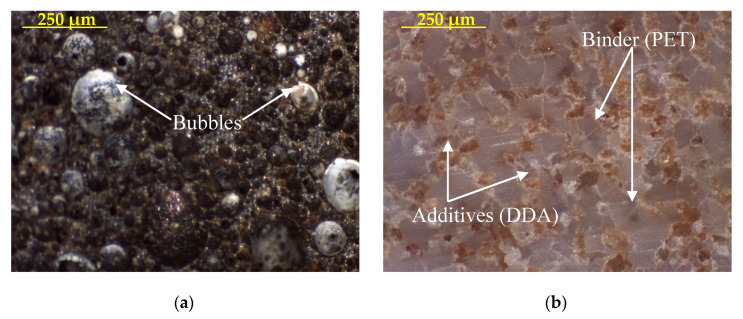

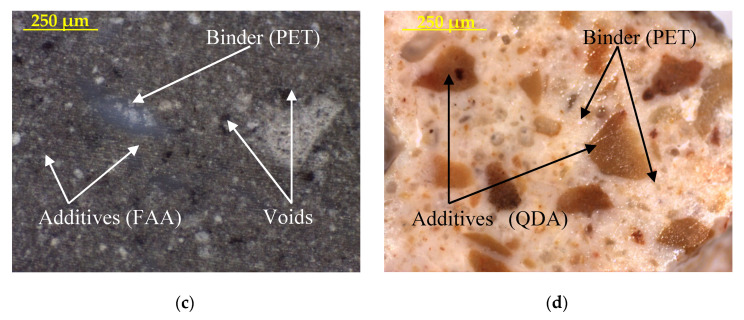
Optical microscopic images of (**a**) volcanic lightweight aggregate (VLA) and plastic based green lightweight aggregates (PGLAs) made with (**b**) dune dust additive (DDA), (**c**) fly ash additive (FAA) and (**d**) quarry dust additive (QDA).

Finally, the strength of aggregate has a direct impact on the concretes’ strength, as the stiffer aggregate provides lower impact value and vice versa [58]. Accordingly, the impact values of NCA, VLA and LYA were 9.65%, 39% and 21.55%, respectively. The impact value of PGLAs was reduced from 42% to 49% and from 1% to 8%, as compared to VLA and LYA, respectively, except PGLA2, which exhibited a marginal increase of 5% as compared to LYA. The high strength of PGLAs (i.e., lower impact value) is likely attributed to good interlocking in the aggregates’ matrix with fewer voids, as shown in the optical microscopic images (see Figure 9).

The optical microscopic images indicate a non-uniform distribution between the plastic waste/binder (PET) and additives (i.e., DDA, FAA, QDA). These images show better bonding between the binder and additives, with fewer voids as compared to that of VLA. Additionally, these images showed the presence of voids in the PGLAs containing FAA (Figure 9c) compared with entrapped air bubbles formed in the VLA, as shown in Figure 9a. Therefore, it is expected that the PGLA concretes would possess a higher strength in comparison with that made from VLA.

Although PGLAs demonstrated a significant reduction in the strength of aggregate (i.e., higher impact value) varying from 105 to 134% in comparison with NCA, the observed impact value of developed aggregate was less than the maximum limit (i.e., 30%) specified by [59]. In addition, concrete made with PGLAs provides a higher strength than that made with VLA, which was also expected due to the better bonding in the aggregate matrix (see Figure 9).

## 5. Green Lightweight Aggregate Concrete Investigation

### 5.1. Fresh Properties

**Figure 10:**
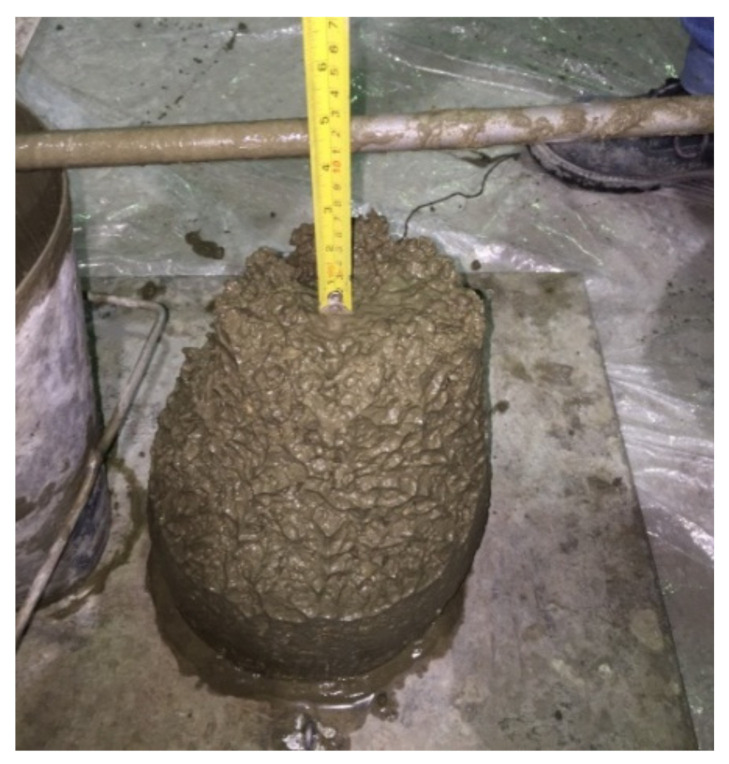
Slump measurement using the cone test method.

The workability of concrete mixes (GLAC, VLAC and NCAC) was measured using the slump cone method (see Figure 10) in accordance with test procedures explained in the ASTM C143/C143M-15a [44]. Table 6 presents a comparison of the slump and fresh density of GLAC (i.e., containing PGLAs) with respect to reference concrete (NCAC, VLAC). As shown in Table 6, the slump of GLAC1 increased by 19%, while the slump of GLAC2 decreased by 40%, as compared to VLAC. In comparison with NCAC, there was a substantial increase of workability of GLACs. The slump of GLAC1, GLAC2 and GLAC3 were increased by 60, 20 and 52.4, respectively, as compared to NCAC. This significant increase is in strong alignment with results, which reported a similar increase varied between 51% and 123% at 75% replacement of NCA with their developed plastic aggregate.

The study results suggest that the maximum slump of GLACs was attained by concretes prepared using PGLA1, which was the coarsest among all developed aggregates; whereas the minimum slump was achieved by that made with PGLA2, which was the finest among all developed aggregates, as reported earlier. This can be linked with the packing level which increases with the finest aggregates and thus reduce the workability, and vice versa. The study results suggest that the concrete made with PGLA2 (i.e., GLAC2) has potential in structural lightweight application, as it fulfils the slump requirements (i.e., 75–125 mm) given in [60].

The fresh densities of concrete mixes containing PGLAs, NCAC and VLAC are presented in Table 6. The fresh density of GLAC1, GLAC2 and GLAC3 were decreased by 13%, 16% and 14%, respectively, as compared to normal weight concrete (NCAC). In comparison with VLAC, the fresh densities of GLAC1 and GLAC3 were marginally increased by 3% and 2%, respectively, whereas GLAC2 achieved a similar density of VLAC. The minimum and maximum fresh densities of concrete made with PGLAs (i.e., GLACs) were achieved by GLAC2 and GLAC1, respectively, because the aggregates used in these mixes (i.e., PGLA2, PGLA1) had the smallest and largest density among all PGLAs (See Table 5).

### 5.2. Dry Density

**Figure 11:**
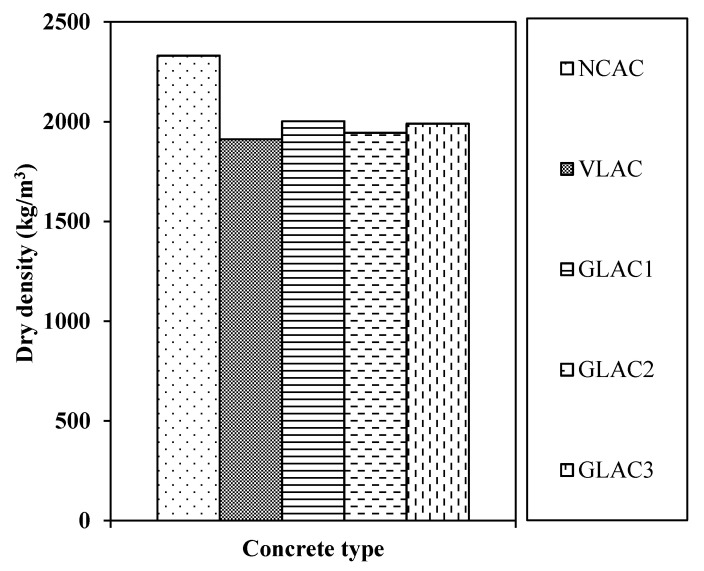
The 28-day dry density of green lightweight aggregate concrete (GLAC), local lightweight concrete (VLAC) and normal weight concrete (NCAC) at W/C of 0.50.

The dry density for concrete made using PGLAs is presented in Figure 11, in comparison with NCAC and VLAC mixes.

Similarly, as observed in fresh density, the dry density of GLACs was slightly higher by 15 to 5% than that of VLAC. However, the dry density of the same mixes was significantly lower (i.e., 14–16%) as compared to that of NCAC. The light weight of the inert materials (i.e., PET plastic waste and additives) used to develop PGLAs was definitely the major source of the density reduction of the developed aggregates (i.e., PGLAs) and its concrete (i.e., GLACs). This decrease is in good agreement with previous studies [15,16,26,27,28]. In those studies, the reduction in dry density varied between 15% and 31% at various replacement levels ranging from 78% to 100% of normal weight coarse or fine aggregate with their plastic aggregates.

Additionally, the minimum and maximum dry densities of GLACs were achieved by GLAC2 and GLAC1, respectively, because the aggregates used in these mixes (i.e., PGLA2, PGLA1) had the smallest and largest density/specific gravity among all PGLAs (see Table 5), as reported previously with fresh density.

Although the dry density of GLACs marginally exceeded the standard limits of [41] (i.e., 1400–1900 kg/m^3^) by 2% to 5%, these findings were in strong alignment with those reported recently by the same author in other studies (density varied from 1906 to 1956 kg/m^3^) [33,34,35,36]. Thus, concrete made with plastic-based green lightweight aggregate has potential in various structural lightweight applications comprising building frames, floors and shell roofs.

### 5.3. Compressive Strength

**Figure 12:**
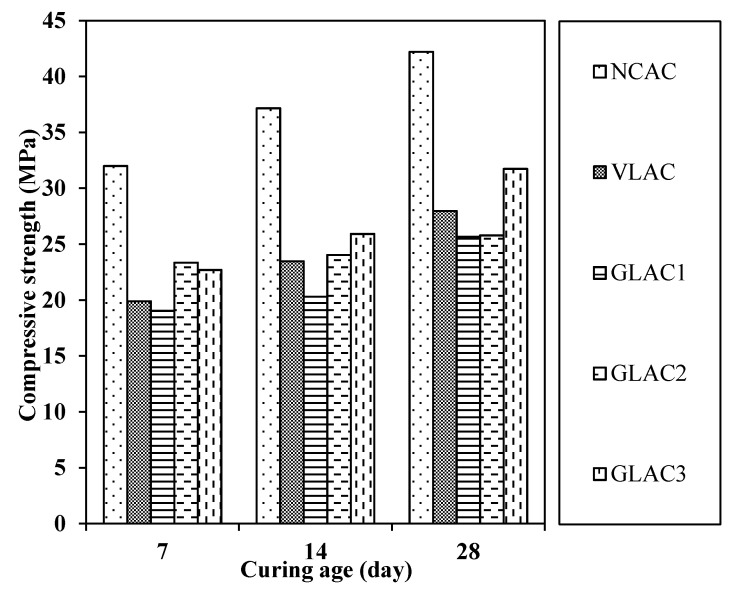
Cube compressive strength (CS) of GLAC, VLAC and NCAC at 7, 14 and 28 days and at W/C of 0.50.

The cube compressive strength (CS) results for GLACs are shown in Figure 12, as compared to NCAC and VLAC at 7, 14 and 28 days, for a W/C of 0.50.

The observation from this figure revealed that the increased percentage in compressive strength for GLACs varied from 3% to 14% as the curing period increased from 7 to 14 days, as compared to 16% and 18% achieved by NCAC and VLAC, respectively. Similarly, the increased percentage in compressive strength for GLACs ranged between 7% and 26% as the curing period increased from 14 to 28 days, as compared to 13% and 19% achieved by NCAC and VLAC, respectively.

At 28 days, the compressive strength of GLACs varied between −8% and 13%, as compared to VLAC. However, in comparison with NCAC, the 28-day compressive strength of GLAC1, GLAC2 and GLAC3 was significantly reduced by 39%, 38% and 24%, respectively. This reduction is less prominent compared to those reported in literature [15,16,26,28,29]. In those works, the compressive strength was reduced from 31% to 66% at various replacement levels that ranged from 75% to 100% of normal weight coarse or fine aggregate with their plastic aggregates. In addition to the low strength of the developed aggregate (i.e., PGLAs) as compared to normal weight aggregate, it is accredited to the decrease in the packing level in concrete mixes, as PGLAs have a relatively poorer grading; which is in agreement with justifications given by [14,30,55].

It worth mentioning that the highest compressive strength was achieved by GLAC3, due to the fact that the strength of the aggregate (i.e., PGLA3) incorporated in this mix was the highest amongst all aggregates (See Table 5). Overall, the 28-day cylinder compressive strength of the GLACs was varied from 20 to 25 MPa; by knowing that the ratio between cylinder to cube compressive strength is 0.8 [61,62]. These findings prove the potential for this developed concrete (i.e., GLACs) to be used in structural lightweight applications, as it was 20% to 49% higher than the lower limit specified in [42] (i.e., 17 MPa) for structural lightweight concrete.

### 5.4. Splitting Tensile Strength

**Figure 13:**
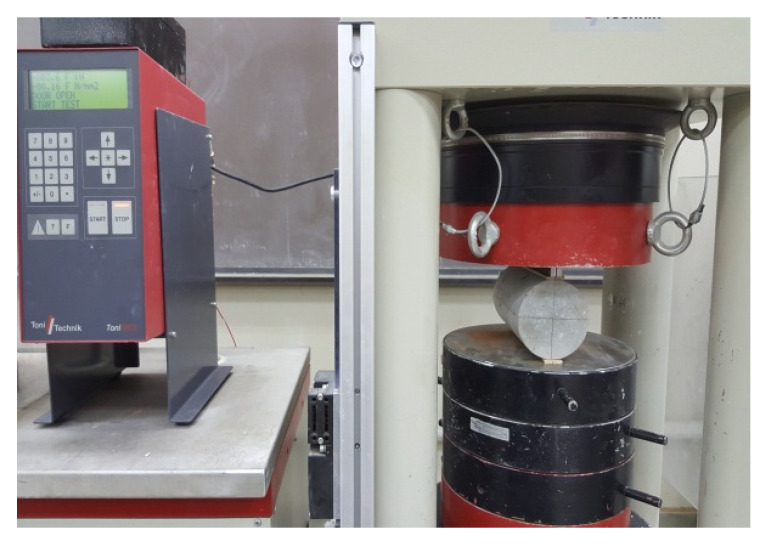
Test setup for splitting tensile strength test.

**Figure 14:**
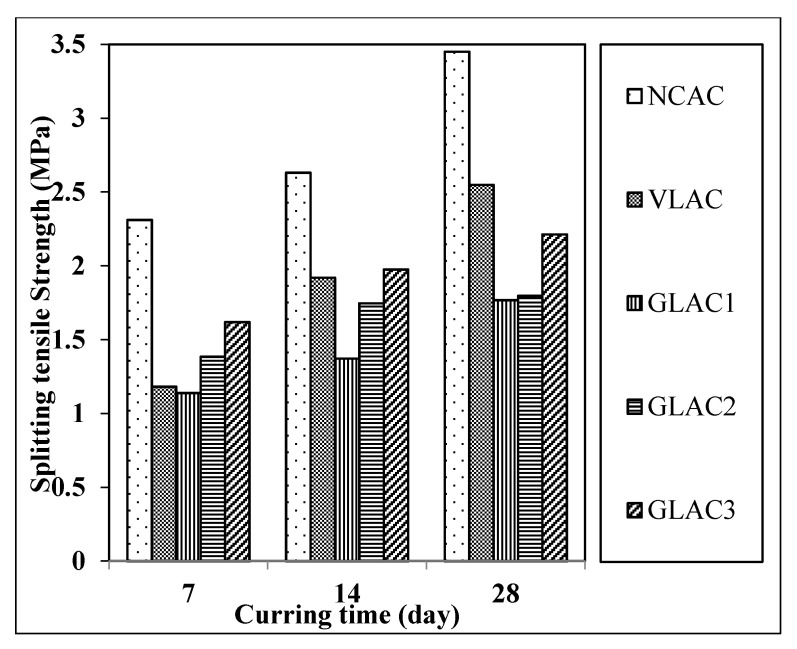
Splitting tensile strength of GLAC, NCAC and VLAC at 7, 14 and 28 days and at W/C of 0.5.

Figure 13 depicts the setup of the splitting tensile strength test for concrete mixes performed in accordance with the test method described in ASTM C496/C496M-11 [48]. The splitting tensile strength results for the concrete mixes made with PGLAs are presented in Figure 14 in comparison with those mixes made with reference aggregates (NCA, VLA). It was observed that the GLACs increased as compared to VLAC at early age but decreased at later ages of 14 and 28 days. In contrast, they were decreased at all ages as compared to NCAC. Therefore, at seven days, the splitting tensile strength of GLACs were varied between −4% and 37% in comparison with VLAC, in which the highest increase was seen in GLAC3 (37%). Conversely, in comparison with NCAC, the splitting tensile strength for the same mixes was lower (maximum reduction observed in GLAC1 by 50%).

However, the 28-day splitting tensile strength of GLACs was significantly reduced from 13% to 30% and from 35% to 48%, as compared to VLAC and NCAC, respectively. These findings are in strong agreement with previous findings reported by [15,16,26]. In those research, the average reduction of the 28-day splitting tensile strength was varied from 31% to 39% at different replacement levels, which ranged from 75% to 100% of normal coarse or fine aggregate with their developed aggregates. This reduction can be explained by the less packing level of the mix, which is due to the aggregates’ poor grading, as mentioned previously (see Section 4).

Overall, these results suggest that the GLAC3 could potentially be used in structural lightweight application since it satisfies the minimum requirements of splitting tensile strength (i.e., 2 MPa) [42], as well as those for compressive strength specified in the same standard method.

### 5.5. Microscopic Investigations and Mechanism of Failure

**Figure 15:**
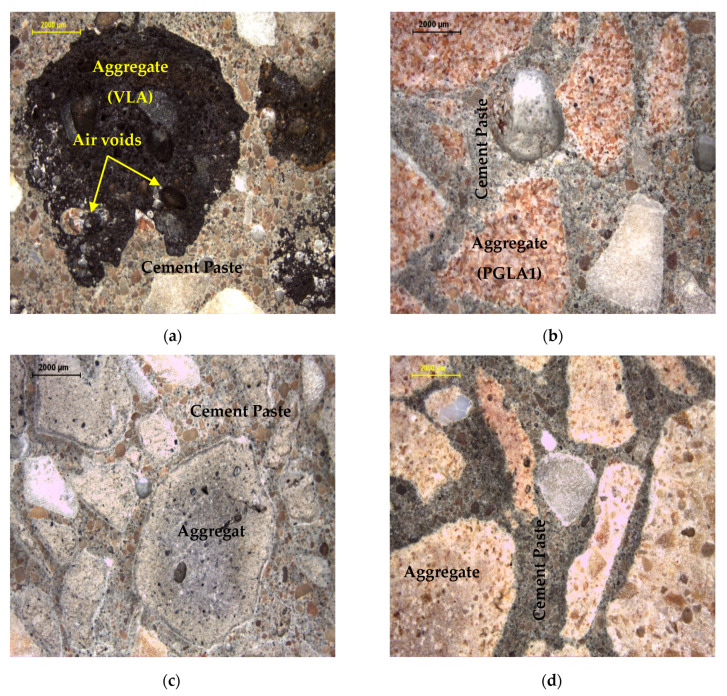
Optical microscopic images of (**a**) VLAC, (**b**) GLAC1, (**c**) GLAC2 and (**d**) GLAC3 (28 days).

The microstructure analysis of green lightweight aggregate concretes (GLACs) was evaluated using an optical microscope at 28 days, as shown in Figure 15.

The main observation of these images indicates that the edges between the aggregates and the cement mortar are clear and well-defined, in contrast with that for VLAC. Additionally, a porous structure with internal air voids was observed for VLA particles (see Figure 15a). This is entirely different to the internal structure of GLACs, which exhibit a solid structure of aggregate particles and perfect interlocking of these particles with the cement matrix, as shown in Figure 15b–d. This observation can be taken as a strong justification for the compressive strength increase for GLAC mixes as compared to that of VLAC. This observation is in strong agreement with [32], who proved that the occurrence of pores in the concrete matrix significantly weaken its bonding and, subsequently, its mechanical properties.

Under compression loading, the mode of failure in GLACs behaved similarly to NCAC, with crack propagation through the cement matrix. In contrast with crack propagation through the aggregate itself observed for VLAC. This conclusion is in a strong agreement with [63], who reported that the rupture occurs through the cement matrix or at weak ITZ for normal weight concrete, since the normal weight aggregate is the strongest part of concrete components. Whereas, in the case of lightweight concrete, the rupture takes place through the lightweight aggregates (i.e., VLA) itself, which is the weakest part of the matrix.

### 5.6. Durability Related Properties

**Figure 16:**
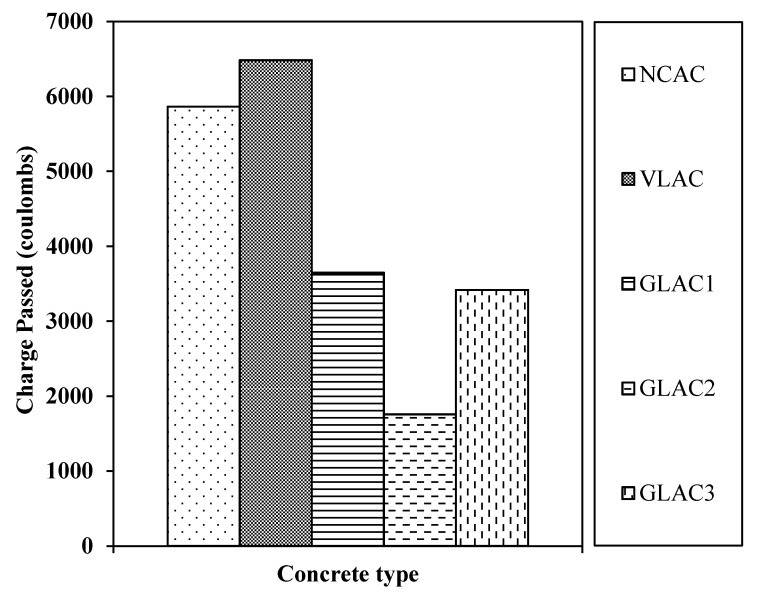
The 28-day chloride permeability of GLAC, NCAC and VLAC at W/C of 0.50.

The resistance of concrete containing PGLAs (i.e., GLACs) towards ingressive soluble (i.e., chloride) was evaluated using a chloride ion penetration test. The results for GLACs are presented in Figure 16, in comparison with NCAC and VLAC.

The general trend shows that the chloride penetration of GLACs was reduced in a range varying from 43% to 72% and from 37% to 70%, as compared to VLAC and NCAC, respectively. The impermeable property of PGLAs particles (i.e., less water absorption) was indeed the main reason for this reduction, which; therefore, distracts the transfer of chloride ions. A similar observation was also reported by [30], who found that chloride permeability reduced by 36% at 45% replacement of fine aggregate with PVC aggregate.

In addition, the least permeability amongst GLACs was attained by GLAC2, which yielded a significant reduction by 72% and 70% in comparison with VLAC and NCAC, respectively. However, the highest permeability was found in GLAC1, which had less reduction by 43% and 37%, as compared to VLAC and NCAC, respectively. This variation in the permeability of GLACs is related to the level of packing in the mix [64], since there were no significant differences in the water absorption amongst the developed aggregate (i.e., PGLAs). Accordingly, the aggregates with the least FM (i.e., less coarse aggregate), such as PGLA2 (see Section 4), achieved a dense level of packing, which ultimately decreases the permeability of the mix. Whereas the coarser aggregates (i.e., with high FM), such as the PGLA1, yield a less dense packing level, which leads to high permeability.

These results reveal that GLACs are more resistant to chloride penetration compared to the control mixes (i.e., VLAC and NCAC). This is due to the closed microstructure (i.e., fewer voids) of the developed aggregate itself (i.e., PGLAs) compared with the relatively high voids accumulated in VLA and NCA, as confirmed by voids value (see Table 5) and microscopic investigation (see Figure 9 and Figure 15).

In summary, these results indicate that the resistance of concrete to chloride ion penetrability increases with the incorporation of PGLAs. These results suggest a potential use for green lightweight concrete in structural and non-structural lightweight applications that are exposed to the risk of chloride attack, since it is classified as having low-to-moderate chloride penetrability according to ASTM C1202-12 [49].

## 6. Conclusions

This study presents the production of plastic-based green lightweight aggregates (PGLAs) containing PET plastic waste and by product additives were developed. The physical and mechanical properties of PGLAs were then compared with those of reference aggregates. Additionally, green lightweight aggregate concrete mixes (GLACs) were produced at 100% replacement of normal weight and lightweight aggregate with developed PGLAs at W/C of 0.5. The fresh, hardened, microscopic and durability-related properties were investigated and compared to those of control mixes. The following conclusions can be drawn from this study:

- PGLAs were successfully developed using PET plastic waste and different types of by product additives. The produced PGLAs presented potential applications for the use in green concrete as a total replacement of conventional VLA and NCA, as they satisfied ASTM C330-04 standard limits. PGLAs exhibit lower water absorption and unit weight together with better grading and high strength.
- Green lightweight aggregate concrete mixes demonstrate high slump due to the shape and texture of the produced aggregates (i.e., PGLAs).
- The unit weight of green lightweight aggregate concretes in fresh and dry state was reduced as compared to normal weight concrete.
- The 28-day compressive strength of green lightweight aggregate concretes was reduced from 24% to 39%, with respect to normal weight concrete; whereas insignificant differences varied between −8% and 13%, as compared to conventional lightweight concrete.
- The 28-day splitting tensile strength of green lightweight aggregate concretes was significantly reduced from 13% to 30% and from 35% to 48%, as compared to lightweight and normal weight concrete, respectively.
- Under flexural loading, the green lightweight aggregate concretes exhibited the same mode of failure for those mixes made with conventional lightweight and normal weight aggregates, as expected. However, under compression loading, the mode of failure in green lightweight aggregate concretes behaved similarly to normal weight concrete, with crack propagation through the cement matrix; in contrast with crack propagation through the aggregate itself, which was observed for conventional lightweight concrete.
- The resistance of concrete to chloride ion penetrability increases with the incorporation of plastic-based green lightweight aggregates. Thus, green lightweight aggregate concrete has potential application in structural and non-structural lightweight applications that are exposed to the risk of chloride attack, as it satisfied the requirements of ASTM C1202-12.
- Green lightweight concrete has the potential to be used in structural and non-structural lightweight applications since it satisfied dry density, compressive and splitting tensile strength requirements specified in ASTM C330. However, these results must be checked in terms of different W/C ratio, chemical admixtures, lone term test and durability test before it can be recommended in a large-scale application.

## Figures and Tables

**Table 1:** Properties of different types of aggregate used in the experimental work.

Test	Coarse Aggregate			Fine Aggregate	
	Normal Weight Aggregate (NCA)	Volcanic Lightweight Aggregate (VLA)	Lytag aggregate (LYA)	Crushed Sand	Red Sand
Bulk Specific Gravity	2.59	1.41	1.44	2.59	2.62
Dry Unit Weight (kg/m^3^)	1554	697	889	1599	1589
Absorption (%)	1.48	18.6	16.82	1.67	0.28
Fineness Modulus	5.83	6.5	-	3.89	1.54
Type	Crushed	Uncrushed	Pelletising	Crushed	Uncrushed
Nominal Maximum Size (mm)	10	10	10	4.75	1.18

**Table 2:** Mix quantities per m^3^ for different mixes.

Concrete Type	Water Cement Ratio (W/C)	Total Water	Free Water	Cement	Fine Aggregates	Coarse Aggregate		
						NCA	VLA	PGLA
		Kg/m^3^						
NCAC	0.5	243.9	228	456	770	784	-	-
VLAC		299	228	456	909	-	352	-
GLAC1		240.8	228	456	708	-	-	637
GLAC2		239.2	228	456	729	-	-	570
GLAC3		242.6	228	456	729	-	-	618

**Table 3:** Standard used for tests conducted on concrete mixes.

Test Type	Testing Age (Day)	Standard Used
Slump test	-	ASTM C143/C143M-15 [44]
Fresh density test	-	ASTM C138/C138M-16 [45]
Dry density test	28	BS EN 12390-7:2009 [46]
Compressive strength test	7, 14, 28	ASTM C579-02 [47]
Splitting tensile strength test	7,14,28	ASTM C496/C496M-11 [48]
Chloride ion penetration test	28	ASTM C1202-12 [49]

**Table 4:** Composition of developed plastic-based green lightweight aggregates.

Sr. No.	Designation	Type of Plastic Waste	Percentage of Plastic Waste	Type of Additives	Percentage of Additives
4	PGLA1	PET	30	DDA	70
5	PGLA2	PET	30	FAA	70
6	PGLA3	PET	30	QDA	70

**Table 5:** Properties of the developed plastic based green lightweight aggregates (PGLAs) as compared to NCA, VLA and LYA.

Property	NCA	VLA	LYA	PGLA1	PGLA2	PGLA3
Bulk Specific Gravity (OD basis)	2.59	1.4	1.44	1.95	1.79	1.94
Bulk Specific Gravity (SSD basis)	2.63	1.67	1.69	1.98	1.81	1.97
Absorption (%)	1.48	18.6	16.82	1.38	1.21	1.68
Dry Unit Weight (kg/m^3^)	1554	697	889	1260	1128	1222
Voids (%)	37.79	50	39.02	24.97	32.82	27.22
Fineness Modulus	5.83	6.5	-	5.87	5.65	5.74
Impact Value (%)	9.65	39.46	21.55	21.33	22.64	19.84
Particles Shape	Angular	Pours	Round	Sub-angular	Sub-angular	Angular
Surface Texture	Rough	Rough	Smooth	Partially rough	Partially smooth	Rough
Colour	White	Black	Brown	Red	Grey	Yellow
Type	Crushed	Uncrushed	Pelletising	Crushed		
Nominal Maximum Size (mm)	10					

**Table 6:** Fresh properties of green lightweight aggregate concrete (GLAC), local lightweight concrete (VLAC) and normal weight concrete (NCAC) at W/C of 0.50.

Sample	Slump (mm)	Fresh Density (kg/m^3^)
NCAC	100	2331
VLAC	210	1968
GLAC1	250	2030
GLAC2	125	1961
GLAC3	210	2013
